# West Nile Virus Viremia in Wild Rock Pigeons

**DOI:** 10.3201/eid1012.040511

**Published:** 2004-12

**Authors:** Andrew B. Allison, Daniel G. Mead, Samantha E.J. Gibbs, Douglas M. Hoffman, David E. Stallknecht

**Affiliations:** * University of Georgia, Athens, Georgia, USA;; † United States Department of Agriculture–Wildlife Services, Athens, Georgia, USA

**Keywords:** viremia, West Nile virus, rock pigeon, host, seroprevelance, dispatch

## Abstract

Feral rock pigeons were screened for neutralizing antibodies to West Nile virus (WNV) during late winter/spring and summer of 2002 and 2003. Additionally, virus isolation from serum was attempted from 269 birds collected during peak transmission periods. The observed viremia levels and seroprevalence indicate that this species could be involved in amplifying WNV in urban settings.

The prototypical amplifying host for most bird-maintained arboviruses, such as West Nile virus (WNV) (*Flavivirus*; *Flaviviridae*), is a species that is locally abundant and readily accessible to arthropod vectors, develops a high level of viremia for an extended duration, and does not develop clinical disease (*1*). Therefore, both the bionomics of the bird species (e.g., population numbers, distribution, association with human habitation/mosquitoes) and its host competence (i.e., susceptibility to infection and ability to circulate virus at titers high enough to infect vectors) need to be evaluated when assessing whether it may be important in amplifying WNV (*1*). Historically, potential host competency for WNV has been determined through experimental infections (*2**–**4*) and, accordingly, supporting viremia levels from free-ranging birds to validate such laboratory-derived competence indices are usually unavailable.

Knowledge regarding the potential host competency of most North American bird species for WNV is limited. In a recent study, Komar et al. experimentally determined WNV viremia levels for 25 bird species encompassing 17 families, whereby an index for reservoir competence was calculated based on the susceptibility of each species to infection, the mean daily infectiousness, and the duration of infectious viremia (*5*). As all species tested were susceptible to infection, the calculated reservoir competence was therefore inherently dependent on the magnitude and duration of viremia. Species that had viremia levels of <10^5^ PFU/mL were considered to be noninfectious for two enzootic vectors, *Culex pipiens* and *Cx. quinquefasciatus*, and hence deemed incompetent hosts. Rock pigeons (*Columba livia*) were included in this group.

Rock pigeons are distributed throughout the entire continental United States and are a gregarious and abundant species, especially in urbanized areas. Field studies have demonstrated that this species has high seroprevalence rates (*6**–**8*) and therefore may be useful as a sentinel to monitor WNV transmission. Additionally, rock pigeons are nonmigratory, which allows for a more accurate determination of approximate sites of exposure than nonresident species. The objectives of this study were to assess the extent of natural infection in free-ranging rock pigeons from metropolitan Atlanta 1 and 2 years subsequent to the recognition of WNV in Georgia and to field-validate experimental results relating to potential levels of viremia in this species.

## The Study

During February, March, and August 2002 and April, July, and September 2003, rock pigeons from northwest Atlanta rail yard, Fulton County, Georgia (33°48´40.1´´N, 84°27´28.4´´W) (Figure), were collected by Wildlife Services personnel by using rocket nets as part of a cooperative nuisance wildlife removal project. Captured birds were identified as hatch-year or adult before being bled by brachial venipuncture for serum collection. A subset of these birds was transferred to captivity as part of an unrelated study. Serum samples collected during late winter/spring (February–April) were frozen at –70°C until screening for antibodies by a plaque-reduction neutralization test (PRNT). Serum samples collected during summer transmission periods (July–September) were tested for circulating virus before being frozen at –70°C until further processing (see below). From 8 separate collections, 499 pigeons were sampled during the 2-year period.

**Figure:**
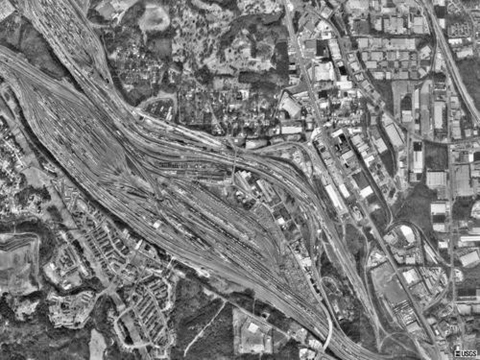
Effect of tularemia and anticancer chemotherapy on the lymphocyte counts and antibody response in a patient with gastric cancer.

WNV antibody titers were determined by PRNT (*6*), with the following modifications. Infected Vero Middle America Research Unit (MARU) cell cultures were overlaid with 1% gum tragacanth/1x minimum essential media (MEM) (supplemented with 2.2 g/L sodium bicarbonate, 3% heat-inactivated fetal bovine serum, 200 units/mL penicillin, 200 μg/mL streptomycin, and 500 ng/mL amphotericin B) rather than agarose, and cultures were inactivated on day 4 postadsorption with 10% buffered formalin and stained with 0.25% crystal violet for plaque visualization. Additionally, 100 pigeons from the August 2002 collection were also tested for antibodies to pigeon paramyxovirus-1 (PPMV-1) (*Avulavirus*; *Paramyxoviridae*) by a hemagglutination inhibition (HI) test (*9*).

For statistical analysis, seroprevalence between late winter/spring and summer collections were compared by using a Yates corrected chi-square test (Epi Info version 3.2.2) and 95% confidence intervals were determined according to Newcombe (*10*). Serum samples collected during summer months (July–September) were screened for circulating virus before being frozen at –70°C until titration (positive) or PRNT (negative). Briefly, 5 μL of serum was inoculated into a 2-mL culture of 2-day-old Vero MARU cells and monitored daily for cytopathic effects. WNV isolates were identified by reverse transcription–polymerase chain reaction by using degenerate WNV-specific primers (WN310F, sense primer: 5´-GTSAACAAAACAAACAGCRATGAA-3´; WN686R, antisense primer: 5´-ACWGMTGAYTTYGTGCACCA-3´) that amplify a 376-bp fragment spanning the nucleocapsid and premembrane genes. The Newcastle disease virus (NDV) isolate was identified by using primers directed against the fusion protein gene (sense primer, 5´-CCTTGGTTGAITCTATCCGIAG-3´; antisense primer, 5´-CTGCCACTGCTAGTTGIGATAATCC-3´) (*11*) and further classified as PPMV-1 by monoclonal antibody binding profiles (*12*).

Viral titers of WNV-positive serum samples collected during the summer were determined by plaque assay. Briefly, samples were rapidly thawed from –70°C, and 200 μL of each 10-fold dilution (10^-1^–10^-6^) of serum in MEM was added to duplicate wells of a six-well plate seeded with 4-day-old Vero MARU cells. Adsorption, overlay, and staining procedures were performed as in the PRNT protocol. Dilutions in which 20–100 plaques could be counted (when applicable) were used in determining WNV titers (log_10_ PFU/mL).

WNV-specific antibodies were detected in 128 (25.7%) of 499 rock pigeons tested (Table 1). Overall seroprevalence rates per collection for 2002 were 16%–45% and 11%–50% in 2003. Significant differences in seroprevalence rates were observed between late winter/spring collections (February–April, 37.4%) versus summer collections (July–September, 15.6%) (p < 0.0000001). Of the 133 samples with >90% plaque reduction on the initial screen, 128 were WNV-positive (96.2%), 4 were flavivirus-positive (3.0%), and 1 was St. Louis encephalitis virus (SLEV)–positive (0.8%). Of 269 birds tested for virus isolation, 11 (4.1%) were viremic (Table 2). Viremia levels were 10^2.2^ to 10^7.2^ PFU/mL (mean = 10^4.0^ PFU/mL).

**Table 1:** Flavivirus seroprevalence rates in free-ranging rock pigeons from northwest rail yard, Fulton County, Georgia, 2002–2003^a^

Collection date	No. tested	n (%) WNV+ [95% CI]	n (%) SLEV+ [95% CI]	n (%) FLAVI+ [95% CI]	n (%) viremic [95% CI]
2002					
Feb 28	56	25 (44.6) [32.4–57.6]	0	2 (3.6) [1.0–12.1]	NT
Mar 6–7	107	35 (32.7) [24.6–42.1]	0	1 (0.9) [0.2–5.1]	NT
Aug 15	58	9 (15.5) [8.4–26.9]	0	0	0
Aug 22	68	13 (19.1) [11.5–30.0]	0	0	7 (10.3) [5.1–19.8]
Total	289	82 (28.4) [23.5–33.8]	0	3 (1.0) [0.4–3.0]	7/126 (5.6) [2.7–11.0]
2003					
Apr 16	34	17 (50) [34.1–65.9]	0	0	NT
Apr 29	33	9 (27.3) [15.1–44.2]	0	0	NT
Jul 30	71	8 (11.3) [5.8–20.7]	0	0	2 (2.8) [0.8–9.7]
Sep 5	72	12 (16.7) [9.8–26.9]	1 (1.4) [0.3–7.4]	1 (1.4) [0.3–7.4]	2 (2.8) [0.8–9.6]
Total	210	46 (21.9) [16.8–28.0]	1 (0.5) [0.1–2.7]	1 (0.5) [0.1–2.7]	4/143 (2.8) [1.1–7.0]


^a^WNV, West Nile virus; SLEV, St. Louis encephalitis virus; FLAVI, flavivirus; WNV+, samples in which a fourfold difference in WNV antibody titer over SLEV could be demonstrated; SLEV+. samples in which a fourfold difference in SLEV antibody titer over WNV could be demonstrated; FLAVI+, samples in which a fourfold difference in titer between WNV and SLEV could not be demonstrated and therefore classified as flavivirus positive; CI, confidence interval; NT, not tested for viremia.

**Table 2:** West Nile virus (WNV) viremia titers of free-ranging rock pigeons from northwest rail yard, Fulton County, Georgia, 2002–2003

Bird ID no.	Collection date	Log_10_ PFU/mL
3309^a^	8/22/2002	2.3
3316	8/22/2002	5.3
3325	8/22/2002	4.4
3494	8/22/2002	3.4
3498	8/22/2002	3.5
3518	8/22/2002	3.3
3524	8/22/2002	2.2
4025	7/30/2003	4.4
4070	7/30/2003	4.4
4288	9/5/2003	3.6
5206^b^	9/5/2003	7.2

^a^Hatch-year bird; all other viremic pigeons were identified as adults. ^b^Died from a pigeon paramyxovirus-1 infection 11 days postcapture; virus identification of serum isolate as WNV was confirmed by reverse transcription–polymerase chain reaction of extracted RNA from serum and neutralization assays using Newcastle disease virus and WNV-specific antisera.

## Conclusions

In 2002–2003, we conducted a serologic study on WNV exposure rates of rock pigeons from a single locale adjacent to metropolitan Atlanta. Consistent with previous studies documenting high WNV exposure rates in this species (*6**–**8*), overall seroprevalence rates per collection for 2002 were 16%–45% and 11%–50% in 2003. The seasonal discrepancy in seroprevalence between late winter/spring collections (37.4%) versus summer collections (15.6%) may be partially ascribed to the influx of naïve juveniles into the population during months of quiescent or reduced virus activity before the onset of peak transmission in late summer.

Of 269 birds tested for virus isolation, 11 (4.1%) were viremic. Since viremic birds were provisionally identified by cell culture, the lag time from serum collection to virus isolation did not afford daily screening for subsequent serum titers. Thus, we cannot delineate the daily mean titer, maximum titer, or duration of viremia for any of these birds. With an overall average WNV viremia titer of 10^4.0^ PFU/mL, our findings are similar to the daily mean titers (10^2.9^–10^4.3^ PFU/mL) of rock pigeons reported in experimental infections (*5*). However, while the maximum titer seen experimentally (10^4.8^ PFU/mL) was below the inferred threshold necessary to infect *Cx. pipiens* and *Cx. quinquefasciatus* (10^5.0^ PFU/mL), 2 of 11 (18%) naturally infected birds had titers in excess of this threshold.

Of note, the rock pigeon with the highest WNV viremia titer (10^7.2^ PFU/mL) became ill 8 days postcapture and died within 72 hours of the onset of clinical signs. PPMV-1, an antigenic variant of NDV, was subsequently isolated from brain and heart tissue. PPMV-1 was not detected in serum. Whether the high-level WNV titer in this viremic pigeon was influenced by coinfection with PPMV-1 (or an undetected pathogen) or whether the level is normal and may occur under natural conditions cannot be determined. Although the effects of WNV coinfection with most microbes and parasites remains unknown, antibodies to PPMV-1 were detected in 68% (n = 100) of the birds tested, and numerous additional pathogenic viruses, bacteria, protozoa, fungi, and helminths have been isolated from free-ranging rock pigeons (*13*). These findings suggest that multiple concomitant infections may occur with regularity in feral populations.

Rock pigeons are intimately associated with urbanization, such that stable populations do not exist outside of human development. Although accurate U.S. population numbers are not available, censuses from various North American cities have estimated urban densities to be in range of 11.4 to 30.8 birds/km^2^ (*14*). This number would equate to a rock pigeon population of 1.7–4.6 million for a city the size of Atlanta. As rock pigeons are ubiquitous in all cities and towns throughout the United States, they could potentially provide an abundant host for enzootic/epizootic vectors such as *Cx*. *tarsalis* and *Cx*. *quinquefasciatus*, both of which have been shown to preferentially bloodfeed on columbiforms (*15**,**16*).

Apart from the study by Komar et al., which, because of its extensive scope, only included six pigeons for viremia determinations, detailed species-specific experimental and field studies assessing the competency of common, urbanized bird species for North American strains of WNV are lacking (*5*). Although the overall viremia titers obtained from naturally infected birds corroborate previous experimental reports that rock pigeons generally maintain low-level viremia titers in relation to passerine species (*2**–**5*), there were outliers that exhibited titers sufficient to infect engorging mosquitoes. This finding exemplifies the need, as duly noted by Komar et al., that experimentally derived competence indices should be consolidated with field data to better estimate host potential (*5*). To our knowledge, this is the first report of viremia levels from wild birds naturally infected with WNV.
